# Georgia State Dental Society

**Published:** 1889-10

**Authors:** 


### Georgia State Dental Society.

The twenty-first annual meeting of the Georgia State Dental Society was held at Tybee Island, in the mouth of the Savannah river, June 11th to 15th, 1889.

The attendance was large, the papers good, and largely devoted to the discussion of pathological conditions. The discussions were animated and to the point.

The President, Dr. S. A. White, of Savannah, the Corresponding Secretary, Dr. L. D. Carpenter, of Atlanta, and the Treasurer, Dr. H. A. Lowrance, of Athens, were promptly on hand. The Recording Secretary, Dr. H. H. Johnson, being on the other side of the Atlantic, "trying his luck" in London, Eng., Dr. D. D. Atkinson filled his place very acceptably. The Vice-Presidents were also conspicuous by their absence.

The president, on the score of recent severe illness, was excused from the customary annual address.

Dr. W. C. Wardlaw, Augusta, Chairman of the Committee on Physiology and Etiology, read a paper entitled

ETIOLOGY OF CARIES.

The paper reviews briefly the list of secondary causes, as nationality, heredity, the influence of sex and systemic disorders, the malformation of individual teeth, etc., with a passing glance at the different theories advanced in the past, all of which have been supplanted by the more recent given theory, of which a brief, popular, but comprehensive *resume* was given. In brief, firmentation of organic matters in contact with the teeth, is caused by bacteria, which generates lactic acid, by which the teeth are decomposed. We are indebted for our present knowledge on the subject to the painstaking and laborious investigations of many scientific men, conspicuous among whom stands Dr. Miller, of Berlin, who being a practical dentist, devoted himself to the study of the bacteria of the mouth with especial reference to their agency in caries.

Microbes were found in both the saliva and carious dentine in great numbers and in great varieties. Saliva and carious dentine were sterilized and no fermentation took place; microbes were introduced and fermentation began at once; when an antiseptic was added no fermentation ensued. The culture solution being impregnated with carious dentine, sound dentine was introduced and decomposition resulted, having all the characteristics of natural caries. Gradually there was isolated a particular species of microbe always found in caries, and which, by itself alone, was able to cause decay. A certain acid was always found present with this micrococcus, which further investigation proved to be lactic acid, a natural product of the microbe of caries and the element decomposing the lime salts of the teeth; pressing forward in advance it breaks down the walls of tubuli, the micrococci follow, crowding in; softening and breaking down proceeds in all directions, and we have a typical case of caries. Whether artificially produced in sound dentine, introduced into the impregnated culture solution, or in the mouth; wherever are found the conditions of heat and moisture, a lodging place for particles of food which are taken possession of by bacteria, these secreting lactic acid, which dissolves to inorganic elements of the tooth substance, giving ingress to other bacteria which consume the organic elements.

The practical teachings of the paper are—first, that absolute freedom of the teeth from the contact of fermenting matter will positively prevent decay; second, that fermentation can be prevented or arrested by the use of antiseptics; third, that bacteria must be destroyed by germicides; fourth, that lodging places for bacteria must be hermetically sealed with materials not subject to their influence so as to prevent their entrance.

The essayist also concludes that a number of cavities of decay are so many hot beds for the propagation of innumerable micro-organisms which communicate caries, by infection, to teeth which might otherwise remain sound. It is also evident that it is not the acids introduced into the mouth with food and fluids that cause decay; also that the state of the saliva, whether neutral, alkaline, or acid, makes but little difference except as it may modify the fermentation due to bacteria. This also explains the ortherwise puzzling inconsistency of the simultaneous presence of caries and incrustations of tartar.

The views of the essayist met with such general endorsement and acceptance that its discussion by Drs. Catching, Adair, Browne, Holland, White, Carpenter, McElhaney and others, brought out no dissenting views. As Dr. R. B. Adair said—as we cannot all be microscopists we must accept the evidences laid before us by those who have demonstrated it to themselves, and who have thrown a flood of clear light on what has hitherto been such an obscure subject.

Dr. S. A. White does not accept the idea of infection from one tooth to another, but that each cavity is dependent upon itself alone and its own germs. He considers bichloride of mercury as a dangerous agent for use in the oral cavity, but for the past fifteen years has relied, with satisfactory results, on the following mouth-wash, used on a soft tooth brush:

½ oz. carbolic acid crystals.
3 oz. glycerine.
3 oz. water.

He is satisfied from his records that those who use this faithfully will require less dental work than others.

In closing the discussion, Dr. Wardlaw said that his attention had first been seriously drawn to the subject by the display of photo-micrographs at Louisville, and the remarks of Dr. Sudduth, but that the more he studied it, the more convinced he became that it is a theory which meets all requirements; it admits of mathematical and chemical demonstration; all other causes being eliminated, we find everything here. It is not claimed, however, that lactic acid does everything; mineral acids help in the work of abrasion, and in affording a *point d'appui* for the micrococci, which penetrate, thence forming the cavity. They must, however, have a protected point of lodgment, as lactic acid requires twenty-four hours for perfection of action. As to the question of "infections," the germs are floating around in the fluids of the mouth ready to lodge wherever they find a good place.

As Chairman of the Committee on

PATHOLOGY AND THERAPEUTICS

Prof. John H. Coyle read a paper considering certain conditions of the oral cavity, which are practically the cause of the loss of the teeth by caries and otherwise. The germ theory being accepted, there are, nevertheless, certain conditions of the secretions of the oral cavity which act as a first cause. An active acid reaction dissolves out a portion of the lime salts of a tooth, producing a local habitat for the specific microbes, which produce caries. The real pathological significance of this condition is too often insufficiently appreciated. This acid condition is due to various causes, among others, local inflammation or irritation of the gums; the cause of this irritation is immaterial, the result being invariable. Wherever there is a disturbance in nutrition, there is a corresponding disturbance in secretion. Notably in pregnant women there are functional disturbances, which result in the most intense and persistent acid condition of the oral fluids, the most perfectly inserted and scrupulously finished gold fillings being sometimes literally washed out and the teeth left perfect wrecks. More time and care should be directed to curative and prophylactic measures for this specific condition. The principle is in all cases the same—*remove the cause.* If due to pregnancy, endeavor to induce an alkaline condition of the secretions.

For this purpose, Dr. Coyle prescribes 15 grs. bi-carb. potassa in cinnamon water, fifteen minutes after each meal. During a period of many years he has failed to learn of a single case where this simple formula has failed of so modifying this condition, as to apparently divest it of any great power of a deleterious character on the teeth.

In the brief discussion which followed the reading of this paper, Dr. Wardlaw said that he was very glad to find that so many were engaged in studying pathological conditions. He thought the prescribed methods of the essayist the very thing most needed. In his own practice he prescribes the free use of lime water as a mouth-wash.

Dr. R. B. Adair, Chairman of the Committee on Anatomy and Surgery, read a paper on the

SURGICAL TREATMENT OF ALVEOLAR ABSCESS,

Being a clinical report of several cases of successful surgical treatment of obstinate chronic cases that had resisted the usual methods of treatment through the tooth. In addition to the surgical treatment from a point external to the apex of the root, the cavity was, in each case, syringed thoroughly with equal parts peroxide of hydrogen and bichloride of mercury, 500 per cent. solution, with an occasional substitution of one drop of creosote.

The subject was discussed by Drs. Holland, Coyle, E. F. Adair, S. A. White, Carpenter, Browne, McElhaney, Eagle Boozer, D. D. Atkinson, and others. Drs. Holland and Coyle described cases in which cure was had only through extraction, amputation of necrosed portion of root, and replantation. In reply to questions, the essayist states that he makes an incision in the gum through which he smooths and cleans off the diseased root and removes all necrosed bone, pus and debris.

Dr. White finds that in ninety-nine cases out of a hundred, one thorough injection of pure creosote and iodine will be found sufficient. Fill the root, and at the end of a week no trace of fistula will be found. For the removal of necrosed bone, nothing equals a sharp burr in the engine; there is no mistaking the touch of sound bone; it is like cutting cheese and coming to the rind.

Dr. Adair said that only simple, recent cases would yield to the once pumping-through process; when due to necrosis of the alveolus or absorption of the root, surgical interference was indicated and essential.

Dr. L. D. Carpenter thought members should be more cautious in saying any operation was "impossible;" better say "in my hands it is not a success." Twenty years ago he had a case in which the nerves of the four superior anterior teeth in which the nerves were dead and gone, the bone so honey-combed that injections through any one tooth would pass through all of the others; the teeth had been in this condition for ten years. They were treated thoroughly with pure wood creosote pumped through and filled the same day. The operation in this case was a success as there has never been any return of discharge. Unless when there is constitutional or hereditary complication, he is successful in such cases with the help of Nature and a kind Providence.

Dr. Holland reported a similar case of the six front teeth, of eight years standing.

Dr. Boozer said that examination of the pus would usually show the condition and cause and indicate the treatment. When there are diseased tissues, which nature, unaided, cannot throw off, this is indicated in the character of the pus discharge.

In closing the discussion, Dr. R. B. Adair said that "in his hands" it was as impossible to cure a certain class of cases at one sitting, as to fly. When there is extensive destruction of tissues with sharp, jagged edges, with the root absorbed, they will not heal without the surgical removal of all diseased tissue.

Dr. Adair described his method of regulating, showing models of several interesting cases, with the appliance used. For rotating he uses a platinum band on which is soldered a little cylinder; in this is slipped one end of a coil of wire, fine piano wire; the other is flattened and passed between the teeth. He prefers ligatures or the spiral spring to jackscrewes, etc.

Dr. White uses the same shaped plate for every kind of irregularity—a vulcanite plate with a band run on the outside of the incisors and covering the bicuspids and molars; there is also a band on the palatine surface. He never allows a metallic plate in contact with the natural teeth; he runs blocks or wedges of rubber between the plate and the teeth where needed, which exert sufficient pressure without ligatures.

Dr. Colding said that, though it might stamp him as behind the times, he still preferred the jackscrew in many cases.

Dr. Browne protested against the huge plates so often used in regulating. A simple band with cylinders soldered to it, and ligatures, would accomplish everything with little discomfort to the patient.

Dr. Holland objects to the jackscrew and piano wire as exerting a continual strain, with no rest. With the wedge, nature has time to build up and recruit and there is less danger of inflammation.

Dr. Adair said that in all cases a retaining plate must be worn until the teeth are held perfectly solid by matured bone deposits.

The Committee on Operative Dentistry had no paper to present. Dr. D. D. Atkinson desired a discussion on the subject of copper amalgam, and reported a case of discoloration of a tooth, apparently from the use of a screw post of copper wire; the tooth was perfectly translucent and of the color of green glass; it had a handsome gold filling and was perfectly preserved, but the color was very objectionable. On the lip was an ulcer fitting the tooth as closely as the hull of a nut. This healed, leaving a blue-black cicatrix after the removal of the filling and screwpost, the tooth being temporarily filled with gutta percha.

The discussion brought out a number of similar cases, all seemingly due to the same cause. The discoloration was found very difficult to remove permanently.

Dr. G. W. McElhaney read a paper on

MECHANICAL DENTISTRY,

The discussion of which failed to elicit anything new.

For those who wear velvet and fine linen, continous gum is undoubtedly the best material, to which nothing else can be compared in point of cleanliness and beauty; but to the poor man, rubber is a blessing, and, when properly manipulated, capable of rendering good service—in comparison far beyond the mere difference in cost.

Dr. Thompson thinks the electro-metallic deposit work, destined to work a revolution in this branch. Dr. Thompson spoke very strongly in favor of celluloid when pressed in dry heat between metallic surfaces, using plain teeth. The color is good, the material is light, and when properly manipulated, durable and free from shrinkage, and capable of giving the most artistic results in gum contour and restoration of features.

Dr. Carpenter considers celluloid one hundred per cent. better than rubber, the latter producing catarrhal, bronchial and throat affections, which are often cured by the substitution of a celluloid for a red rubber plate.

Dr. White prefers gold but makes a mere skeleton plate—a gold wire or band not more than a quarter of an inch wide, fitting the mouth perfectly, but not allowed to come in contact with any remaining natural teeth, considering it very objectionable to cover the roof of the mouth.

Dr. Catching offered a verbal report from the Committee on Dental Education and Literature. He spoke of standard literature, periodical literature, college institutions, and association work, as the four great methods of professional education. Standard literature is the basis and foundation by which the profession is judged; periodical literature keeps a man abreast with the rapid progress of the profession; association work is another source of elevation too often ignored by the busy practitioner.

The post graduate school is the latest and greatest step in advance. He regretted the failure of exhibitors to include in their displays samples of the literature of the profession, which he thought should be made a prominent feature.

Dr. H. S. Colding read a paper on Practical Education, taking the ground that the dentist is born, not made by the college, dentistry being, in his view, two-thirds mechanics and one-third common-sense, medicine and surgery. To prepare a student for the dental college, he would put him under a first class machinist for two or three years, and then give him a summer in the dental infirmary and laboratory under competent demonstrators; not until then would he be prepared to profit by the course of lectures of the college proper.

Dr. B. H. Catching read a paper entitled "Some Things To Do," embodying a number of practical points in little things.

Dr. W. C. Browne read a similar paper entitled "A Few Points Confirmed by Experience."

The following officers were elected:

President, S. B. Barfield, Macon; 1st Vice-Pres., R. B. Adair, Gainesville; 2nd Vice-Pres., W. G. Browne, Atlanta; Rec. Sec., C. A. Rider, Gainesville; Cor. Sec., L. D. Carpenter, Atlanta; Treasurer, H. A. Lowrance, Athens,

Gainesville was chosen as the next place of meeting, the second Wednesday in July, 1890.

